# The effect of a single blood meal on the phenotypic expression of insecticide resistance in the major malaria vector *Anopheles funestus*

**DOI:** 10.1186/1475-2875-7-226

**Published:** 2008-10-31

**Authors:** Belinda L Spillings, Maureen Coetzee, Lizette L Koekemoer, Basil D Brooke

**Affiliations:** 1 Vector Control Reference Unit, National Institute for Communicable Diseases, NHLS, Private Bag X4, Sandringham, 2131, South Africa; 2 Division of Virology and Communicable Disease Surveillance, School of Pathology of the National Health Laboratory Service and the University of the Witwatersrand, Johannesburg, South Africa; 3 NRF Chair in Medical Entomology and Vector Control, School of Pathology, University of the Witwatersrand, Johannesburg, South Africa

## Abstract

**Background:**

*Anopheles funestus *is a major malaria vector in southern Africa. Vector control relies on the use of insecticide chemicals to significantly reduce the number of malaria vectors by targeting that portion of the female population that takes blood meals and subsequently rests indoors. It has been suggested that the intake of a blood meal may assist female mosquitoes to tolerate higher doses of insecticide through vigour tolerance. It is hypothesized that during the process of blood digestion, detoxification mechanisms required for the neutralizing of harmful components in the blood meal may also confer an increased ability to tolerate insecticide intoxication through increased enzyme regulation.

**Methods:**

Bottle bioassays using a range of concentrations of the pyrethroid insecticide permethrin were performed on pyrethroid susceptible and resistant laboratory strains of *An. funestus *in order to detect differences in insecticide susceptibility following a single blood meal. Based on these results, a discriminating dosage was identified (double the lowest dosage that resulted in 100% mortality of the susceptible strain). Blood-fed and unfed females drawn from the resistant strain of *An. funestus *were then assayed against this discriminating dose, and the percentage mortality for each sample was scored and compared.

**Results:**

In the insecticide dose response assays neither the fully susceptible nor the resistant strain of *An. funestus *showed any significant difference in insecticide susceptibility following a blood meal, regardless of the stage of blood meal digestion. A significant increase in the level of resistance was however detected in the resistant *An. funestus *strain following a single blood meal, based on exposure to a discriminating dose of permethrin.

**Conclusion:**

The fully susceptible *An. funestus *strain did not show any significant alteration in susceptibility to insecticide following a blood meal suggesting that vigour tolerance through increased body mass (and increased dilution of internalized insecticide) does not play a significant role in tolerance to insecticide intoxication. The increase in insecticide tolerance in the pyrethroid resistant strain of *An. funestus *following a blood meal suggests that insecticide detoxification mechanisms involved in insecticide resistance are stimulated by the presence of a blood meal prior to insecticide exposure, leading to enhanced expression of the resistance phenotype. This finding may be significant in terms of the methods used to control indoor resting populations of *An. funestus *if the mass killing effect of insecticide application proves increasingly inadequate against blood-feeding females already carrying the insecticide resistance phenotype.

## Background

Malaria is the most prevalent vector borne disease worldwide, and predominantly affects third world and developing countries [1]. Many of these countries experience limited economic growth, which is, in part, exacerbated by the effects of malaria. The impact of malaria can be seen by decreased levels of productivity in home and work environments and by the added strain that this disease places on already overburdened health care systems [2].

The two major malaria vectors in southern Africa are *Anopheles arabiensis *and *Anopheles funestus. Anopheles gambiae *can be found in the northern-most parts of this sub-region [3]. Malaria in South Africa is hypoendemic. The primary malaria vector, *An. funestus*, is controlled by an indoor residual spraying (IRS) campaign that currently adopts a mosaic approach using DDT (dichloro-diphenyl-trichloroethane), carbamates and pyrethroids [4]. This approach was adopted after pyrethroid resistance was identified in South African and southern Mozambican *An. funestus *populations [5,6]. Pyrethroid resistance in this species was closely associated with a dramatic increase in malaria incidence in South Africa during the period 1995 – 2000. Prior to this period, only DDT was used for IRS, but mounting international pressure to discontinue its use led to the implementation of pyrethroids as the insecticide of choice [7]. DDT was re-introduced for IRS in South Africa post 2000 and a five- to six-fold decrease in malaria incidence was recorded for the period 2001 to 2005 [4]. The development of insecticide resistance in other malaria vector populations in South Africa [8] induces additional cause for concern, and an understanding of the mode, expression and inheritance of insecticide resistance mechanisms has become increasingly important.

Insecticide resistance in insect populations is predominantly based on improved enzymatic sequestration and detoxification as well as by the alteration of insecticide target sites leading to insecticide insensitivity [9]. Improved enzymatic detoxification has been linked to three broad classes of enzymes, namely monooxygenases, glutathione-S-transferases (GSTs) and non-specific esterases. Pyrethroid resistance in *Culex quinquefasciatus *[10], *Culex pipiens pipiens *[11], *An. gambiae *[12] and *An. funestus *[6,13] has been linked to the increased activity of cytochrome P450s, members of the monooxygenase class of detoxification enzymes.

The P450 monooxygenases are a superfamily of enzymes that have been implicated in the detoxification of xenobiotics and endogenous metabolic products in insects [14]. Clusters of cytochrome P450 genes have been associated with pyrethroid resistance based on the chromosomal mapping of quantitative trait loci or QTLs [15,16]. In southern African *An. funestus*, a QTL associated with pyrethroid resistance can be found on chromosome 2R. This position corresponds to the locality of a cluster of genes belong to the CYP6 class of P450 enzymes [15]. CYP6P9, located within this cluster, has been shown to be highly over-expressed in a pyrethroid resistant strain of *An. funestus *[13], confirming the importance of these enzymes in insecticide resistance.

Current evidence suggests a direct link between the increased expression of detoxification genes and the development of pyrethroid resistance in southern African *An. funestus *[13]. Since many major biological processes affect gene expression it is possible that insecticide detoxification gene expression may be stimulated by processes other than insecticide exposure. The upregulation of cytochrome P450s in response to a blood meal has been demonstrated in *C. pipiens *[17] and *Aedes aegypti *[18]. It is hypothesized that the detoxification of xenobiotics and toxic blood components in the *An. funestus *midgut may inadvertently result in an increased ability to tolerate insecticide intoxication.

## Methods

### Mosquito strains

*Anopheles funestus *colonies have been established and are maintained at the Vector Control Reference Unit of the National Institute for Communicable Diseases, NHLS (Johannesburg, South Africa). All colonies are maintained under standard insectary conditions [19]. The two *An. funestus *strains used were: Fumoz-R, which originates from southern Mozambique and has been intensively selected for pyrethroid resistance [19], and FANG, which originates from Angola and is susceptible to pyrethroids.

### Insecticide dose-response experiments

The process of blood meal digestion may activate detoxification systems required to detoxify xenobiotics present in the blood. Hence, it was decided that insecticide susceptibility should be investigated at different stages during the blood digestion process. The early stage of digestion was investigated four hours post blood meal based on the assumption that those genes involved in the digestion process would have been expressed by that time. The later stage of the digestion process was investigated at 18 hours post blood meal to allow for the possibility that different genes may have been upregulated by that time.

Three to four day old female cohorts from each strain were collected. Each cohort was divided into two groups, one fed on 10% sucrose solution and the other to be blood-fed. Blood meals were offered in a darkened room with an ambient temperature of 25°C. Only females that took blood were subsequently tested for susceptibility to permethrin. Following blood-feeding, a 10% sucrose solution was made available to all the females for either four hours or 18 hours prior to permethrin exposure.

Dose-mortality responses comparing blood-fed versus unfed samples from the permethrin resistant and susceptible *An. funestus *colonies were assayed according to the CDC bottle bioassay method [20]. Glass bottles (250 ml volume) were coated with the following range of permethrin concentrations (μg of permethrin/250 ml bottle): 0.1 μg, 1 μg, 10 μg, 25 μg, 50 μg, 100 μg, 250 μg, 500 μg and 1000 μg. Appropriate amounts of permethrin (Sigma catalogue 45614) were dissolved in 1 ml acetone as a carrier. Each bottle was used a maximum of three times before being discarded.

Each series of insecticide exposures lasted one hour, following which all females were transferred to polystyrene cups with access to a 10% sucrose solution. Percentage mortality was recorded 24 hours post-exposure for each permethrin concentration. For each *An. funestus *colony, eight to twelve cohorts were used. The mean percentage mortality was calculated at each insecticide dose and the dose response curves plotted. The data was log transformed to allow for the calculations of the 50% lethal dose (LD50) value for each replicate of each cohort, using regression analysis. The mean LD50 and standard deviation could then be calculated for blood-fed and unfed, resistant and susceptible mosquitoes.

### Dose specific responses following a blood meal

The WHO defines the discriminating dosage of insecticide to be used in resistance assays as twice the amount of insecticide required to kill 100% of an insecticide susceptible sample of the same species [21]. The susceptible FANG strain showed 100% mortality at approximately 50 μg/250 ml bottle. It was thus decided that investigations at 100 μg/250 ml bottle would be appropriate for dose specific assays against Fumoz-R. Insecticide dosages of 2 μg and 5 μg/250 ml bottle were chosen for dose specific assays against the susceptible FANG strain based on results from the dose-response experiments where the range induced approximately 50% mortality.

Three to four day old female cohorts from each strain were removed and divided into two groups: one for blood-feeding and one to be fed on 10% sucrose solution. Blood meals were offered four hours prior to the one hour permethrin exposures. Twenty to twenty five females were exposed per bottle through 9 to 11 replicates. Final mortality was recorded 24 hours post exposure and comparisons between blood-fed and unfed groups for each *An. funestus *strain were based on 2 sample *t*-tests and one-way ANOVA.

## Results

### Lethal dose response curves

**Figure 1:**
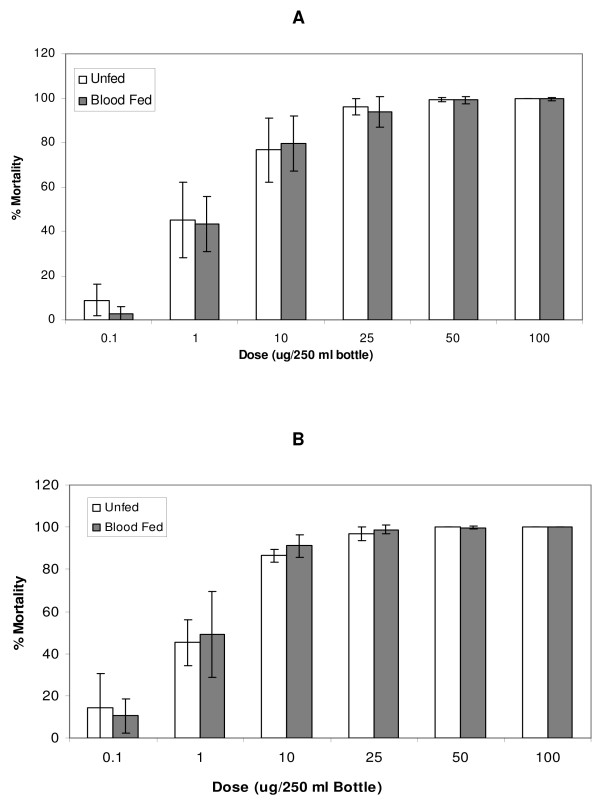
Percentage mortalities 24 h post exposure for the pyrethroid susceptible *An. funestus *strain (FANG), in response to permethrin exposures, with either (A) blood-feeding 4 hours prior to permethrin exposure or (B) blood-feeding 18 hours prior to permethrin exposure.

**Figure 2:**
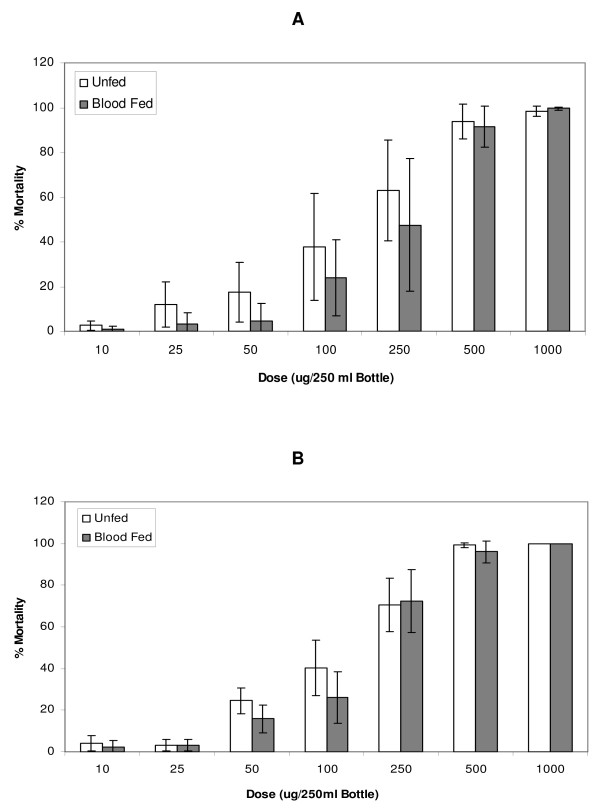
Percentage mortalities 24 h post exposure for the pyrethroid resistant *An. funestus *strain (Fumoz-R), in response to permethrin exposures with either (A) blood-feeding 4 hours prior to permethrin exposure or (B) blood-feeding 18 hours prior to permethrin exposure.

Dose response curves were generated for the insecticide susceptible FANG (Figure 1) and permethrin resistant Fumoz-R (Figure 2) strains. No significant difference in susceptibility to permethrin between the unfed and blood-fed groups for both Fumoz-R and FANG (p > 0.05) was evident across the full dosage range, regardless of the lapse of time between blood-feeding and permethrin exposure. However, the Fumoz-R strain showed consistently higher levels of permethrin tolerance in the blood-fed group as compared to the unfed.

**Figure 3:**
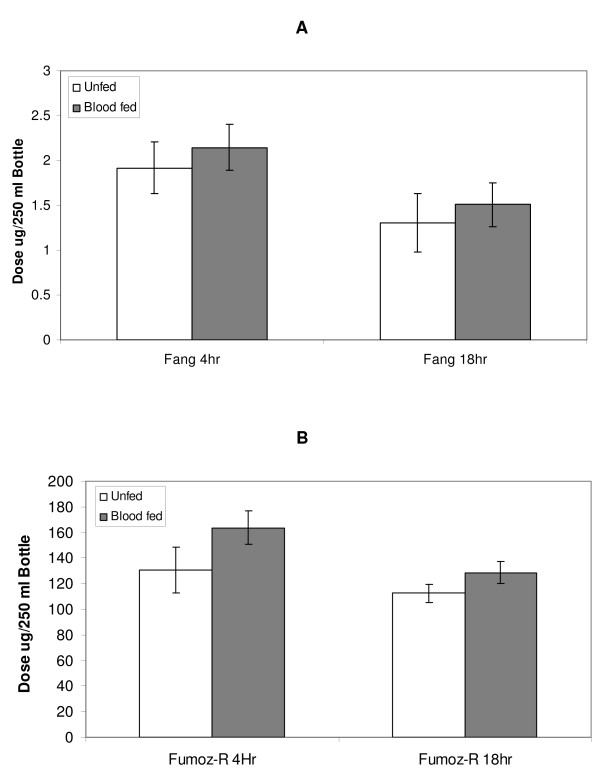
**Comparison of dosages required to produce 50% mortality, 24 h post permethrin exposure, (A) in the susceptible *An. funestus *strain (FANG) and (B) the resistant *An. funestus *strain (Fumoz-R). **Permethrin exposures were carried out at either 4 hours or 18 hours post blood-feeding.

Figure 3 shows the dose of permethrin required to produce 50% mortality in each of the strains, for each of the treatment times (permethrin exposure at either 4 hours or 18 hours post blood-feeding). Fumoz-R showed significantly higher levels of insecticide tolerance as compared to the susceptible FANG strain (p < 0.05). The permethrin dose required to kill 50% of the resistant Fumoz-R samples was approximately 70 to 80 times greater than that for the susceptible FANG strain.

### Dose specific responses following a blood meal

**Figure 4:**
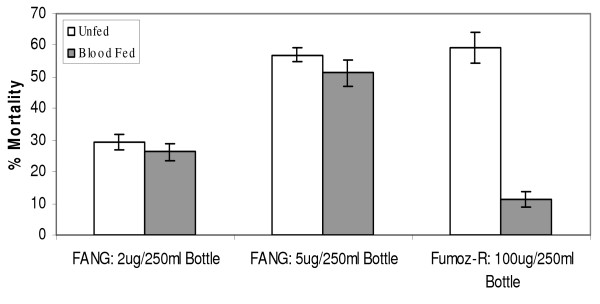
**Comparison of percentage mortalities 24 h post permethrin exposure at chosen discriminating dosages for the susceptible (FANG) and resistant (Fumoz-R) *An. funestus *strains.** Permethrin exposures were carried out 4 hours post blood-feeding at 2 and 5 μg/250 ml bottle for FANG and 100 μg/250 ml bottle for Fumoz-R.

The susceptible FANG strain showed no significant difference in response to permethrin exposure between the unfed and blood-fed groups, for both of the insecticide dosages tested (p > 0.05, Figure 4). The difference in response to permethrin exposure between blood-fed and unfed cohorts from the Fumoz-R strain was highly significant (p < 0.001) with the blood-fed cohorts showing a mean percentage mortality approximately five times lower than that of the unfed cohorts (Table 1).

**Table 1:** Mean percentage mortalities at the discriminating dosages for the susceptible *An. funestus *strain (FANG) and the resistant *An. funestus *strain (Fumoz-R).

	**Dose/250 ml Bottle**	**Mean % Mortality**	**SE**	**n**	**p**
**FANG Unfed**	2 μg	29.45%	2.57%	213	> 0.05
**FANG Blood-fed**	2 μg	26.31%	2.80%	216	

**FANG Unfed**	5 μg	57.00%	2.42%	261	> 0.05
**FANG Blood-fed**	5 μg	51.33%	4.14%	246	

**Fumoz-R Unfed**	100 μg	59.21%	5.01%	244	< 0.001
**Fumoz-R Blood-fed**	100 μg	11.37%	2.54%	245	

All exposures on blood-fed individuals were carried out 4 hours post blood-feeding. (FANG: pyrethroid susceptible *An. funestus*, Fumoz-R: pyrethroid resistant *An. funestus*.)

"SE" = standard error; "n" = sample size; "p" = significance of difference between the unfed and blood-fed groups following 2 sample *t*-tests.

## Discussion

The development of insecticide resistance in southern African *An. funestus*, and its dramatic effect on malaria transmission in South Africa, has highlighted the need to investigate this phenotype and its controlling factors. Pyrethroid resistance in southern African *An. funestus *has been linked to elevated levels of monooxygenase cytochrome P450 activity as the primary mode of resistance [6,13]. It has subsequently been demonstrated that the resistance phenotype is inherited as a single, autosomal, incompletely dominant genetic factor [22] and that there is no compromise in reproductive and physiological fitness associated with resistance [23], leading to the prediction that pyrethroid resistance can be expected to spread readily within and between *An. funestus *populations in affected areas. If insecticide application is to remain effective, then this scenario must ultimately consider the response to insecticide exposure of older, blood-feeding females, which form that proportion of the population actively transmitting malaria.

The application of an adapted CDC bottle bioassay method [20] allowed for the quantification and comparison of the levels of insecticide tolerance in both insecticide resistant and susceptible *An. funestus *strains, in response to the effect of blood-feeding. The results presented in this study indicate that the permethrin resistant strain (Fumoz-R), which has been intensively selected for pyrethroid resistance, has a 70- to 80-fold increase in insecticide tolerance as compared to the insecticide susceptible strain (FANG). Although the insecticide dose response curves did not highlight any significant differences in insecticide tolerance between any of the blood-fed and unfed cohorts, the blood-fed resistant Fumoz-R strain consistently required higher dosages than its unfed counterpart in order to produce the same level of mortality. The lack of statistically significant differences between blood-fed and unfed cohorts may be an artifact of wide variation in response to insecticide exposure between batches of mosquitoes over successive generations.

The direct comparison of percentage mortality following exposure to discriminating dosages of permethrin showed that a blood meal did not significantly alter the degree of insecticide tolerance in the fully insecticide susceptible strain of *An. funestus*. This result suggests that vigour tolerance through increased body mass (and subsequent increased dilution of internalized insecticide) does not offer a significant measure of insecticide resistance. However, similar comparisons between blood-fed and unfed, insecticide resistant females from the Fumoz-R strain showed a significant increase in insecticide tolerance in association with a single blood meal. This result suggests that the presence of a blood meal combined with an already effective insecticide detoxification mechanism significantly enhances the expression of the resistance phenotype.

Given that IRS campaigns aim to target the biting portion of a vector population that rests indoors and that insecticide resistance phenotypes within *An. funestus *populations are becoming more prevalent, the data presented here warrant further consideration. The results presented here suggest that the presence of a blood meal and/or the process of its digestion activate a series of insecticide detoxification pathways which "prime" the mosquito for contact with insecticide, in all likelihood through the increased expression of P450 genes hypothetically associated with blood meal digestion and insecticide detoxification.

## Conclusion

The fully insecticide susceptible *An. funestus *strain did not show any significant alteration in susceptibility to insecticide following a blood meal suggesting that vigour tolerance through increased body mass did not play a significant role in tolerance to insecticide intoxication. The decrease in insecticide susceptibility in the pyrethroid resistant strain of *An. funestus *following a blood meal suggests that insecticide detoxification mechanisms involved in insecticide resistance are further stimulated by the presence of a blood meal prior to insecticide exposure, leading to enhanced expression of the resistance phenotype. This finding may be significant in terms of the criteria that are used to evaluate resistance phenotypes determined by WHO [21] bioassay in field populations, because blood-fed female mosquitoes may show enhanced expression of the resistance phenotype, possibly allowing for earlier detection of insecticide resistance.

## Competing interests

The authors declare that they have no competing interests.

## Authors' contributions

BDB conceived the project and oversaw the research carried out. He also assisted in data analysis and preparation of the manuscript. BLS carried out the laboratory work and data analysis, wrote the first and subsequent drafts of the manuscript. MC and LLK contributed to the project design and assisted in the writing of the manuscript.

All authors have read and approved the final manuscript.
